# Known mechanisms cannot account for a third of reduced susceptibility in non-*aureus* staphylococci

**DOI:** 10.1038/s44259-023-00008-1

**Published:** 2023-11-13

**Authors:** Heather Felgate, Lisa C. Crossman, Elizabeth Gray, Rebecca Clifford, Annapaula Correia, Rachael Dean, John Wain, Gemma C. Langridge

**Affiliations:** 1 Medical Microbiology Research Laboratory, Norwich Medical School, University of East Anglia, Norwich Research Park, Norwich, NR4 7UQ UK; 2 Quadram Institute Bioscience, Norwich Research Park, Norwich, NR4 7UQ UK; 3 School of Biological Sciences, University of East Anglia, Norwich Research Park, Norwich, NR4 7TJ UK; 4 SequenceAnalysis.co.uk, Norwich Research Park Innovation Centre, Norwich, NR4 7JG UK

**Keywords:** Bacterial genetics, Bacterial genetics, Population genetics

## Abstract

Non-*aureus* staphylococci (NAS) are implicated in many healthcare-acquired infections and an understanding of the genetics of antimicrobial resistance is important in relation to both clinical intervention and the role of NAS as a reservoir of resistance genes. Gap statement: The burden of antimicrobial resistance in NAS, particularly to clinically relevant antimicrobials, is under-recognised. We sourced 394 NAS isolates from clinical samples, healthy human volunteers, animals and type cultures and subjected them to minimum inhibitory concentration (MIC) testing by agar dilution using eight antimicrobials. Cefoxitin was used to screen for methicillin resistance, as it stimulates the expression of *mecA* in *S. aureus*. We performed whole genome sequencing on 366 isolates and analysed these genotypically for the presence of genetic mechanisms responsible for the phenotypic levels of reduced antimicrobial susceptibility. We observed 175 sequenced isolates with a MIC ≥ 4 µg/ml to cefoxitin, of which 50% did not harbour a known *mec* homologue. Eight clinical NAS isolates displayed high daptomycin MICs (>4 µg/ml), with no known mechanism identified. Differences in MICs against erythromycin were attributable to the presence of different resistance genes (*msrA* and *ermC*). In total, 49% of isolates displayed reduced susceptibility to three or more of the antimicrobials tested. The widespread presence of reduced antimicrobial susceptibility in NAS is concerning. An increased likelihood of harder-to-treat infections caused directly by NAS with acquired resistance genes has clinical implications for AMR detection, the horizontal resistance gene pool and the management of patients.

## Introduction

The non-*aureus* staphylococci (NAS) represent an important source of nosocomial disease, including prosthetic joint infection (PJI), infective endocarditis and infection in pre-term babies^1^. In the UK, over 215,000 joint replacements (hip, knee and shoulder) took place in 2016, with a year-on-year increase of 4%^2^. Of these replacements, 1.5% require surgical revision due to infection^2^. These infections are most commonly caused by *Staphylococcus* spp., and attributed to NAS in ~31% of cases across Europe^3^. In our local hospital, the Norfolk and Norwich University Hospital (NNUH), 50% of isolates identified in suspected PJI are NAS.

In clinical microbiology, staphylococci are classified using the coagulase test, with coagulase-positive samples overwhelmingly identified as *S. aureus* and coagulase-negative samples grouped together under the term coagulase-negative staphylococci (CoNS). CoNS is therefore the term found in antimicrobial surveillance data. However, since coagulase-negative *S. aureus* strains exist (as do coagulase-positive strains of other staphylococcal species), we use the term “non-*aureus* staphylococci” (NAS) to encompass all staphylococci which are not *S. aureus*, including the commonly isolated *S. epidermidis*, *S. haemolyticus* and *S. capitis* species, regardless of coagulase activity.

There is currently an intense focus upon the presence and spread of bacterial antimicrobial resistance, typified in *S. aureus* by methicillin resistance (MRSA). While the body of literature in antimicrobial resistance research is growing for staphylococci, NAS data remains eclipsed by the focus on *S. aureus*. Studies investigating antimicrobial resistance (AMR) in NAS^4^ suggest that 45% of NAS harbour methicillin resistance^5^, and that NAS may be resistant to a larger number of antimicrobial classes than *S. aureus*^5,6^ but comprehensive analyses are missing. We aimed to address this point here by curating a diverse collection of NAS and correlating mechanisms of antibiotic resistance with MICs.

## Results and discussion

Our NAS collection comprised over 30 species of *Staphyloccocus*, including at least 10 isolates of *S. epidermidis*, *S. capitis*, *S. haemolyticus*, *S. hominis*, *S. saprophyticus*, *S. simulans* and *S. warneri* (Table 1). Isolates were collected over a 4-year period from 2013 to 2016.

**Table 1:** Frequency of non-*aureus* staphylococcal species^a^ in the study collection.

*Staphylococcus auricularis*	1
*Staphylococcus capitis*	20
*Staphylococcus caprae*	2
*Staphylococcus carnosus*	2
*Staphylococcus chromogenes*	3
*Staphylococcus cohnii*	1
*Staphylococcus condimenti*	1
*Staphylococcus devriesei*	1
*Staphylococcus epidermidis*	191
*Staphylococcus equorum*	1
*Staphylococcus haemolyticus*	43
*Staphylococcus hominis*	45
*Staphylococcus jettensis*	1
*Staphylococcus lugdunensis*	7
*Staphylococcus massiliensis*	1
*Staphylococcus microti*	1
*Staphylococcus muscae*	1
*Staphylococcus nepalensis*	1
*Staphylococcus pasteuri*	4
*Staphylococcus petrasii*	1
*Staphylococcus pettenkferi*	1
*Staphylococcus piscifermentans*	1
*Staphylococcus rostri*	1
*Staphylococcus saprophyticus*	22
*Staphylococcus sciuri*	5
*Staphylococcus simiae*	1
*Staphylococcus simulans*	10
*Staphylococcus* sp. *[1]*	1
*Staphylococcus stepanovicii*	1
*Staphylococcus succinus*	1
*Staphylococcus vitulinus*	3
*Staphylococcus warneri*	18
*Staphylococcus xylosus*	1

^a^Species designated by MALDI-TOF (Bruker).

**Fig. 1:**
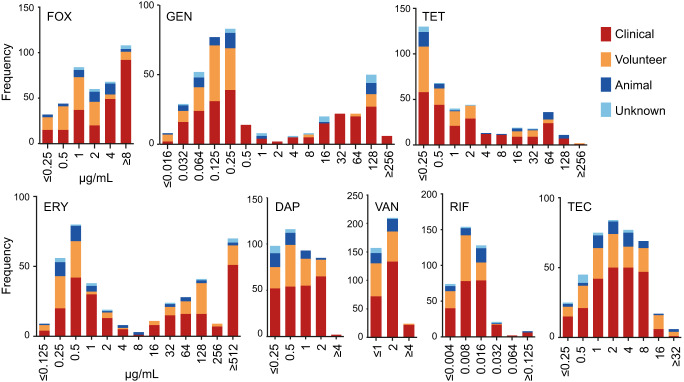
Antibiotic susceptibility of NAS collection. Frequency distributions of MIC values for *n* = 394 NAS isolates grown in the presence of antimicrobials. FOX cefoxitin, GEN gentamicin, TET tetracycline, ERY erythromycin, DAP daptomycin, VAN vancomycin, RIF rifampicin, TEC teicoplanin.

The range of antimicrobials tested was selected based on clinical relevance and availability (Supplementary Table 1). Observed MIC distributions per antimicrobial are shown in Fig. 1 which demonstrates how susceptibility varies within this NAS collection. Erythromycin, tetracycline and gentamicin all displayed bimodal distributions, with erythromycin indicating an additional population of very high MICs. The other five antibiotics displayed Gaussian distributions.

### Cefoxitin screening does not correlate with *mecA* presence in clinically relevant NAS

**Fig. 2:**
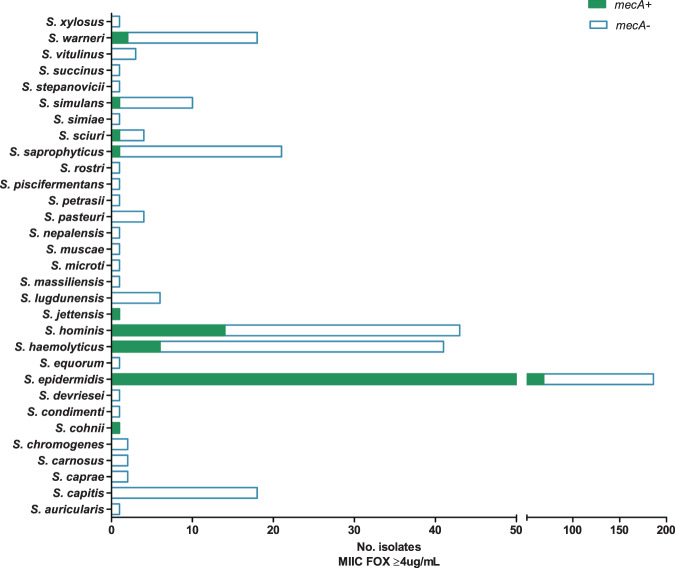
Presence of *mecA* in relation to high cefoxitin MIC. Per staphylococcal species, bars display the total number of sequenced isolates found to have a cefoxitin MIC of ≥4 µg/ml, in relation to the presence (solid green) and absence (blue outline) of *mecA*.

Cefoxitin is used to screen for methicillin resistance in *S. aureus* as it induces *mecA and mecC* expression^7^. However, while methicillin-resistant *S. aureus* (MRSA) has a high public profile, much less is known about methicillin-resistant NAS (MRNAS). EUCAST guidelines state that for MRSA “cefoxitin is a very sensitive and specific marker of *mecA/mecC*-mediated methicillin resistance including in heterogeneous expressing strains and is the agent of choice”^8^. In this collection, we found 194/394 (49%) displayed reduced susceptibility to cefoxitin with MICs ≥ 4 µg/ml (Supplementary Table 1). The vast majority of these isolates were from clinical samples (FOX Fig. 1, Supplementary Table 1) but analysis at the nucleotide level (Supplementary Tables 2 and 3) indicated that only 88 out of 175 (50 %) sequenced isolates with an MIC ≥ 4 µg/ml harboured a known *mecA*. MecA is extremely well-conserved and analysis at the amino acid level yielded the same results (Supplementary Table 4). Other *mec* elements were also identified (e.g. *mecC*, *mecI* and *mecR1*) but only ever in addition to *mecA*. Breaking this down by species, 20/21 *S. saprophyticus* isolates with cefoxitin MIC ≥ 4 µg/ml harboured no *mecA* (Fig. 2). No *mecA* was detected in eleven species with high cefoxitin MICs and for *S. hominis*, *S. warneri* and *S. haemolyticus*, the percentage of the population that exhibited MIC ≥ 4 µg/ml with no *mecA* was between 28% and 67% (Fig. 2). Our results support cefoxitin as a good indicator of *mecA* presence in *S. epidermidis*^9^, but suggest that it performs poorly in the less common, but still clinically relevant NAS. In addition, we observed 14 cases where the presence of *mecA* did not result in a MIC ≥ 4 µg/ml. These were re-tested (alongside 6 others) under conditions designed to encourage *mecA* expression (see the “Methods” section) and resulted in increased MICs in all isolates, however, 7/20 remained <4 µg/ml (Supplementary Table 1). An alternative mechanism for high FOX MICs could be beta-lactamase production, however, *blaZ* was the only beta-lactamase detected in the collection, and there were >30 isolates where high FOX MICs were observed in the absence of *mecA* and *blaZ* (Supplementary Table 2).

### Reduced susceptibility only partly explained by known mechanisms

When exposed to gentamicin and tetracycline, isolates could broadly be divided into two populations, displaying susceptible or reduced susceptibility phenotypes (Fig. 1 GEN and TET). In isolates displaying an MIC ≥ 1 µg/ml for gentamicin, 49/130 isolates harboured *aac(6’)-le-aph(2”)-la* (Table 2) which is associated with gentamicin resistance in *Enterococcus*^10,11^ but has also been observed in *Staphylococcus*^12,13^. A total of 12 isolates had a match for *aph(3’)IIIa*, but only five of them were associated with reduced susceptibility.

**Table 2:** Genetic mechanisms identified using ARIBA/ABRIcate and the CARD database compared to the MIC data (of sequenced isolates only, partial and interrupted sequences are not included, see Table S2).

Antimicrobial	Mechanism (Accession No.)	No. isolates above breakpoint MIC (≥2 µg/ml)	No. isolates below breakpoint	
TET	*tetK* (NC_013452)	48/148	5/223	
	*tetL* (M11036.0)	1/148	0/223	
	*tetM* (AM180355)	1/148	0/223	
	All^a^	50/148 (33.8%)	5/223 (2.2%)	
		**No. isolates above breakpoint MIC (≥1** **µg/ml)**		
GEN	*aac(6’)-Ie-aph(2”)-Ia* (NC_005024)	49/130	6/246	
	*aph(3)IIIa* (CP004067)	5/130	7/246	
	All^a^	49/130 (37.7%)	12/246 (4.9%)	
		**MIC** **≥** **512** **µg/ml**	**MIC** **≥** **2–256** **µg/ml**	
ERY	*msrA* (NC_022598.1)	6/66	75/135	9/173
	*ermC* (M12730)	42/66	13/135	9/173
	*ermA* (NC_009632)	6/66	1/135	0/173
	*emeA* (AB091338)	1/66	0/135	0/173
	All^a^	46/66 (69.7%)	88/135 (65.2%)	18/173 (10.4%)

*TET* tetracycline, *GEN* gentamicin, *ERY* erythromycin.

^a^Some isolates harboured multiple resistance genes.

**Fig. 3:**
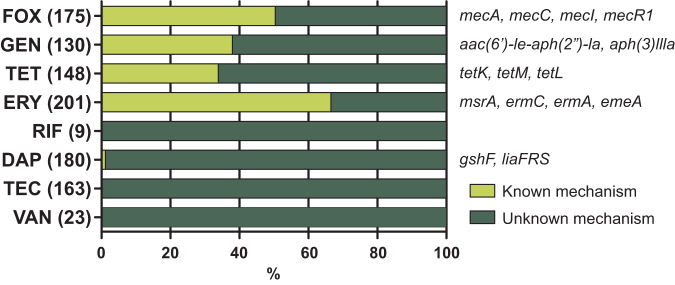
Mechanisms of resistance. Percentage of known genetic mechanisms identified in sequenced NAS isolates with reduced susceptibility. Number of isolates with reduced susceptibility per antimicrobial is given in parentheses. Known mechanisms found per antimicrobial are shown in italics. FOX cefoxitin, GEN gentamicin, TET tetracycline, ERY erythromycin, DAP daptomycin, VAN vancomycin, RIF rifampicin and TEC teicoplanin.

Six isolates that contained *aac(6’)-le-aph(2”)-la* displayed susceptible MICs, making them the equivalent of major errors (MEs) in public health terms, as the isolates were genotypically resistant but phenotypically susceptible^14^. Accordingly, the 81/130 isolates with reduced susceptibility (≥1 µg/ml) that harboured no *aac(6’)-le-aph(2”)-la* represented the equivalent of very major errors (VMEs) as they were genotypically susceptible but phenotypically resistant^14^. This is highly suggestive of novel mechanisms of resistance and was a feature of other antimicrobials tested (Fig. 3). It is possible that assessing antibiotic susceptibility on agar may have produced different MICs. This is mitigated by two aspects: firstly, our study design incorporated 13.5% replication in the MIC assay giving additional confidence to the results as all repeats were within 2-fold MIC, and secondly, even if the MIC value per isolate was different on agar, the spread of MICs would be highly likely to remain and require mechanisms to explain reduced susceptibility.

To identify whether efflux pumps might play a role in these phenotypes, we assessed the ARIBA output for the staphylococcal-specific *norABC, mgrA, mepR* and *qac* genes^15^. In the sequenced NAS collection, 168/378 (44.4%) contained *norA*, however of these less than two-thirds (75/130) had reduced susceptibility to gentamicin.

According to the Comprehensive Antimicrobial Resistance Database [CARD]^16^, *tetK* is by far the most common tetracycline resistance mechanism in *S. aureus* and *S. epidermidis* (10–20%), followed by *tetL* (<1%) and *tetM* (<1%). This was borne out in our NAS collection, where 48/148 (32.4%) isolates with MICs ≥ 2 µg/ml of tetracycline contained *tetK*, as compared to 5/223 (2.2%) with MICs below 2 µg/ml. One animal isolate with an MIC of 16 µg/ml carried *tetL* and one clinical isolate with an MIC of 64 µg/ml carried *tetM*; neither had any other tetracycline resistance genes. Again, this demonstrated that 98/148 isolates displayed a reduced susceptibility phenotype that did not associate with a known resistance determinant, indicative of uncharacterised resistance mechanisms.

The distribution of erythromycin phenotypes was more complex. With this antimicrobial, we observed both susceptible isolates and those with reduced susceptibility, but the latter appeared to consist of two populations, one with MICs between ≥2–256 µg/ml and one with MICs ≥ 512 µg/ml (Fig. 1 ERY). We had sequence data available from 135 of the ≥2–256 µg/ml population and 66 of the ≥512 µg/ml population, and identified the presence of a resistance gene (*ermA, ermC, msrA)* in 65.9% (89/135) of the ≥2–256 µg/ml population and 55/66 (83.3 %) of the ≥512 µg/ml population (Table 2). Our results indicated that the presence of *ermC* rather than *msrA* was the major cause of MICs exceeding 256 µg/ml. Although rare (*n* = 4), harbouring both genes resulted in a MIC ≥ 512 µg/ml in three cases and 128 µg/ml in the other. In isolates with a MIC of ≥ 512 µg/ml, *qac* was identified 36 times. In 23 of these cases, *ermC* was also present; *qac* was only found twice with no other known erythromycin resistance mechanisms present. A total of 101 isolates with a MIC ≥ 2 µg/ml did not contain *qac*.

For daptomycin, approximately half the collection displayed reduced susceptibility (MIC ≥ 1 µg/ml, Fig. 1 DAP and Supplementary Table 1). A small subset, comprising eight isolates from clinical samples only, displayed MICs ≥ 4 µg/ml; such high MICs to daptomycin have not been previously reported, according to the European Committee on Antimicrobial Susceptibility Testing (EUCAST) and The British Society for Antimicrobial Chemotherapy (BSAC) surveillance data. These MICs were repeated a second time and confirmed. This is concerning given that daptomycin is a current therapeutic choice for treating soft tissue infections caused by NAS^17^. Seven of these isolates were sequenced and our ARIBA analysis (Supplementary Table 3) indicated only a single *S. epidermidis* isolate contained genes implicated in daptomycin resistance: *gshF* and *liaFRS* with an MIC of 1 µg/ml, the remaining 169 isolates with an ≥1 µg/ml MIC did not harbour any of these genes. SNP mutations in *mprF* and *rpoC* are associated with daptomycin resistance in *S. aureus* but none of these were identified in the NAS collection^18,19^. More recently, SNPs in *walK* have also been associated with daptomycin resistance in *S. aureus* and *S. epidermidis*^20^. The three *S. aureus* SNPs are present in CARD and were not identified in our collection. The V500F mutation from *S. epidermidis*^20^ was also not identified in our *S. epidermidis* with DAP MICs ≥ 4 µg/ml. Whilst *walK* was identified by protein BLAST as present across the NAS collection (as expected for an essential gene^21^), sequence variation was observed at the protein level which prevents SNPs observed in *S. aureus* or *S. epidermidis* being extrapolated to all NAS. We, therefore, conclude that there are potentially novel daptomycin resistance mechanisms present in these strains.

### Higher MICs found in clinical samples

Vancomycin is a treatment option for prosthetic joint infection, and 94% of isolates had a MIC below 4 µg/ml (Fig. 1 VAN). However, of the 24 isolates with reduced susceptibility, 22 (92%) came from clinical samples and only 2/24 were found in healthy volunteers. This is indicative of a wider trend, where isolates associated with clinical samples had significantly higher MICs (*p* < 0.005) than non-clinical isolates for cefoxitin, erythromycin, gentamicin, tetracycline, daptomycin and vancomycin (Supplementary Fig. s). Given the importance of NAS in nosocomial infections, this is a worrying prospect both in terms of what is present in the clinic and also the possibility of AMR gene transfer into organisms more capable of causing infection, including *S. aureus*. In addition, no known mechanisms of resistance were identified for vancomycin, rifampicin or teicoplanin (Fig. 3 and Supplementary Table 3).

### Over half of the NAS collection displayed susceptibility to multiple antimicrobials

Out of all the isolates tested, 48% (192/394) had reduced susceptibility to three or more antimicrobials. Twenty-five isolates had reduced susceptibility to six antimicrobials, and three isolates had reduced susceptibility to seven antimicrobials; of these 24/25 and 3/3 were isolated from clinical samples (Supplementary Table 1). The implications of these are difficult-to-treat infections and potentially a large reservoir of staphylococcal resistance genes within the patient under antimicrobial treatment.

### Animal isolates have similar MIC distributions to human isolates

It is generally acknowledged that the presence of reduced susceptibility in microorganisms isolated from domesticated animals can impact public health if those organisms also cause infection in humans^22,23^. In our collection, there were 40 NAS isolated from domesticated animals (7 NCTC strains), of which we obtained genome sequences from 23. Animals included were cats, dogs, cattle and sheep and although in much fewer numbers than the human isolates in the collection, the animal isolates displayed very similar MIC distributions and harboured corresponding genetic mechanisms. This does not rule out the possibility that animals could be a reservoir of AMR for staphylococci.

## Conclusion

Genome analysis of isolates displaying MICs to cefoxitin of ≥4 µg/ml indicated that approximately half harboured the *mecA* element. The absence of *mecA* from the other half suggests that other mechanisms are likely present. This was apparent across many of the antimicrobials tested as between 0% and 65% of phenotypic resistance in clinical isolates could be attributed to known resistance mechanisms. The remaining 35–100% suggests that there are potentially numerous unknown mechanisms underpinning NAS resistance, which warrant further investigation.

## Methods

### NAS collection

Under NHS Research Ethics Committee approval, the Norwich Biorepository banks blood, solid tissue and bacterial isolates from the NNUH and research institutes on the Norwich Research Park, including the University of East Anglia (UEA), and makes these available to the research community. This enabled us to assemble a collection of 380 NAS from (a) clinical specimens which were isolated from suspected NAS PJI infections (229, NNUH), (b) healthy human volunteers (114 skin swabs from adults at UEA), and (c) animal samples (33, UEA) with five having no source recorded. Animal isolates were taken from healthy domestic dogs, cats, sheep and cows. An additional 14 strains of NAS from the National Collection of Type Cultures (NCTC) were supplied by Public Health England.

Isolates were identified at NNUH using MALDI-TOF (Bruker) to the species level (Table 1). All strains were cultured overnight on TSA plates (Oxoid), checked for contamination and purified. Once purified, the NAS collection was stored as glycerol stocks to be screened for their antimicrobial susceptibility. *Staphylococcus aureus* NCTC 12973 was used as a control.

### Susceptibility testing

To assay the entire NAS collection, five deep well 96-well microplates (VWR) were prepared with 1 ml TSB (Oxoid) per well. Glycerol stocks were used to inoculate the corresponding well. Per plate, one well was designated as a sterility control (TSB only) and one well was inoculated with the *S. aureus* control. After inoculation, plates were sealed and incubated at 37 ^o^C at 180 rpm for a minimum of 10 h. The experimental design enabled 13.5% of the collection to be tested in duplicate; MIC data were compared and then tabulated (Supplementary Table 1).

Following standard BSAC guidelines (version 14) at the time, iso-sensitest agar (Oxoid) was prepared and sterilised. Antimicrobial stocks were added to obtain the desired final concentrations once the media had cooled to <50 ^o^C. For daptomycin, Ca^2+^ was also added at 50 µg/ml. The agar antimicrobial mixture was then poured into sterile rectangular plates (Fisher Scientific) and dried.

Per strain, a 1:10 dilution of overnight culture was transferred to a 96-well plate and the OD_600_ was measured. An average OD_600_ was calculated for each column, which was then diluted to approximately OD_600_ 0.6 to generate an inoculum plate for susceptibility testing.

Using a 96-pin multi-point inoculator (Denley), ~1 µl of inoculum per isolate was stamped onto the agar containing antimicrobials, from the lowest concentration to the highest. Between inoculum plates, the pins were washed in 70% ethanol for 30 s and allowed to dry before stamping on an antimicrobial-free plate to confirm sterility. Washes were also carried out between antimicrobials using sterile water. All stamped plates were incubated at 37 ^o^C.

Isolates found to have reduced susceptibility to daptomycin had their MICs determined for a second time by spotting 10 μl of culture onto TSA plates containing various daptomycin concentrations (supplemented with Ca^2+^ at 50 µg/ml). To increase *mecA* expression, 14 isolates which contained *mecA* but on initial testing showed susceptibility to cefoxitin (MIC < 4 µg/ml) were re-tested on Mueller Hinton Agar with 3% NaCl added alongside a further 6 isolates. Overnight cultures were diluted in PBS and 5 µl spots containing 10^4^ cells were spotted onto plates which were incubated at 35 ^o^C.

Test MIC ranges (in µg/ml) were as follows: daptomycin 0.25–2, erythromycin 0.125–256, gentamicin 0.016–64, rifampicin 0.004–0.064, teicoplanin 0.25–16, tetracycline 0.25–256 and vancomycin 1–4, cefoxitin 0.25–4 µg/ml (based upon published work^9^). Isolates were considered to have reduced susceptibility to the specified antibiotic if they displayed the following MICs: ≥4 µg/ml (cefoxitin, teicoplanin, vancomycin); ≥2 µg/ml (tetracycline, erythromycin); ≥1 µg/ml (gentamicin, daptomycin); ≥0.06 µg/ml (rifampicin).

### Statistical comparison of clinical and non-clinical isolates

Using Prism (GraphPad, San Diego, USA, v 5.04), a Mann–Whitney test was performed (non-parametric test, two-tailed with Gaussian approximation) to compare the MIC of clinical and non-clinical isolates. Statistical significance was given to a *p-*value < 0.05.

### DNA extraction and sequencing

Overnight cultures derived from single colonies were pelleted and resuspended in lysis buffer (Qiagen), transferred to 2 ml lysis matrix B tubes (MPBio) and subjected to bead beating for 15 min at 30 Hz (Tissuelyser II, Qiagen) with RNAse A added. DNA was extracted according to the QiaCube HT protocol with an additional 30 min incubation at 65 ^o^C after proteinase K addition and eluted into Tris–10 mM HCl.

Libraries for sequencing were prepared using the Nextera XT DNA Library Prep Protocol and sequenced on the Illumina MiSeq or NextSeq with a loading concentration of 1.8 picomolar.

### Genome analysis

The raw reads were subject to FastQC quality control^24^, adapters were trimmed using Trimmomatic [v 0.39]^25^ using the supplied NexteraXT adapter sequences. In some cases, read normalisation was performed using BBNorm [v35.85]^26^ to remove low coverage contamination. The lowest coverage cutoff level parameter used was dependent on the total coverage of the sequence since some sequencing runs had a high difference in coverage level across the run. Finally, reads were concatenated if they originated from Illumina NextSeq since this platform produces eight reads per sample, four forward and four reverse. A total of 364 samples passed QC and were suitable for downstream analysis. These reads were used as described below. Sequences are available from the European Nucleotide Archive, under project PRJEB31403.

To determine which antimicrobial resistance genes and associated individual mutations were present in each of our 364 NAS genomes, reference gene sequences were downloaded from CARD v2.0.0^16^ and used as input to ARIBA v2.13.2^27^ which generates local assemblies from sequence reads and reports back which reference genes (and individual mutations) are identified, with a minimum percent identity cut off at 90% (Supplementary Table 5). For genes where ‘partial’ or ‘interrupted’ was reported, this was not considered sufficient evidence for intact gene presence. The tabulated results were evaluated for gene and mutation presence/absence relative to MIC per antimicrobial. Twelve NCTC sequences were downloaded as genome assemblies from the European Nucleotide Archive (accessions: SAMEA4364213; SAMEA4364214; SAMEA4384234; SAMEA4384058; SAMEA4384237; SAMEA4384064; SAMEA4412661; SAMEA4384059; SAMEA4384235; SAMEA4384060; SAMEA4384339; SAMEA4384403) and analysed by ABRIcate v0.9.7^28^ using CARD v2.0.0^16^ as the reference database with a minimum DNA coverage of 90%. NCTC 13831 and 13837 sequence data was not available at the time of sequencing and therefore these isolates were sequenced as described above for the main NAS collection. Protein level conservation was assessed using BLAST v2.10.1 against the NCBI AMR database. Hits were recorded for greater than 40% identity at the protein level over 80% of the query and subject sequence.

### Reporting summary

Further information on research design is available in the Nature Research Reporting Summary linked to this article.

## Supplementary information

REPORTING SUMMARY

MICs in clinical and non-clinical isolates

Supp Data 1 - MIC characterisation of the NAS collection

Supp Data 2

Supp Data 3

Supp Data 4

Supp Data 5

## Data Availability

Sequences are available from the European Nucleotide Archive, under project PRJEB31403.
