# Supplementary material for: Known mechanisms cannot account for a third of reduced susceptibility in non-aureus staphylococci
Source: NPJ Antimicrob Resist. 2023 Nov 13;1:15. doi: 10.1038/s44259-023-00008-1 (PMC11721661; doi:10.1038/s44259-023-00008-1)
Supplement: Supplementary file 2 — MICs in clinical and non-clinical isolates [file 44259_2023_8_MOESM2_ESM.pdf]

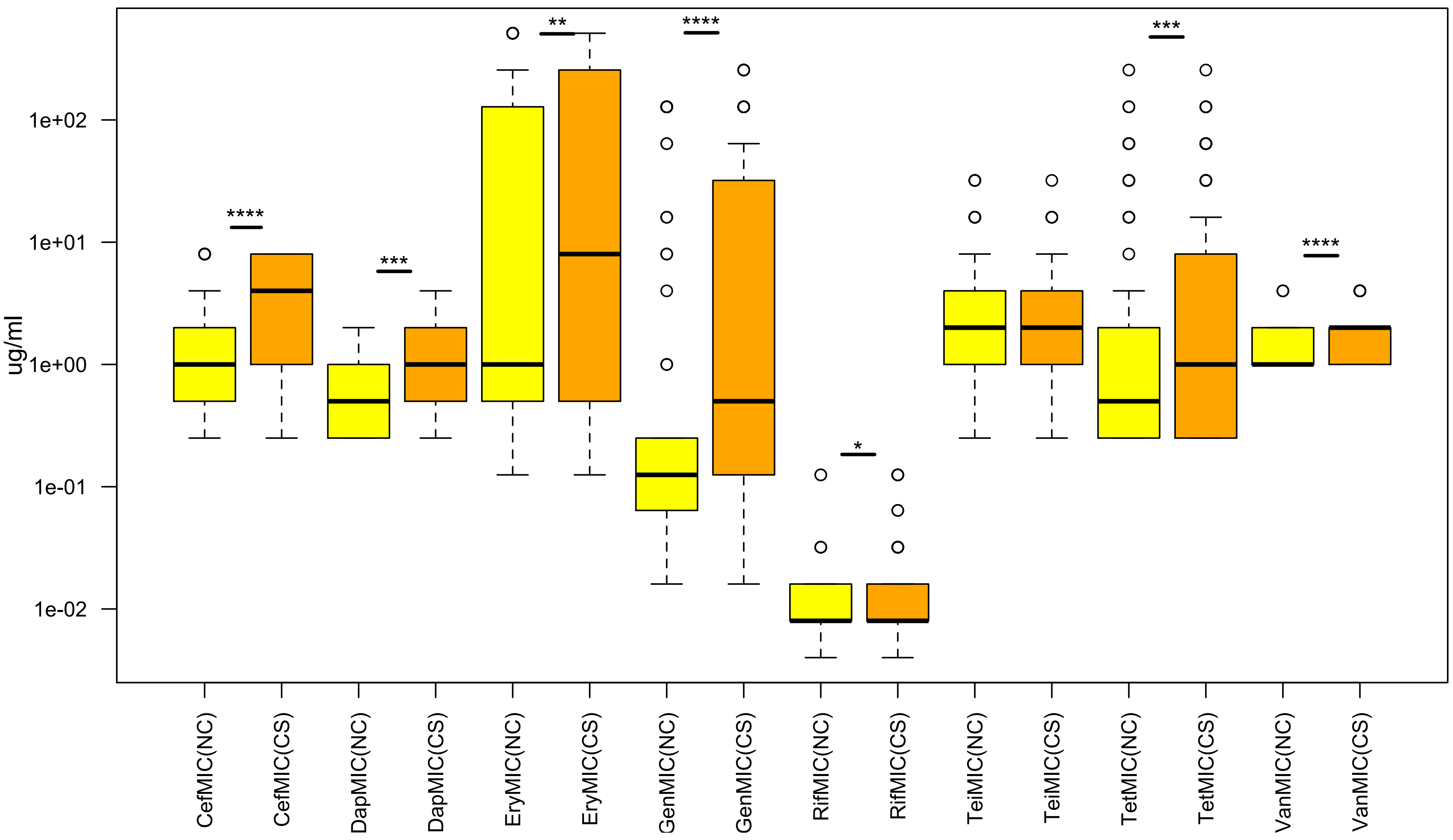

Supplementary Figure 1. MICs in clinical and non-clinical isolates

Box and whisker plot of MIC distribution per antimicrobial for clinical (orange, CS) and non-clinical (yellow, NC) isolates. Thick black bar indicates the median MIC which resided within the Interquartile Range. The extreme lines are represented as dotted lines and indicate the data outside the upper (75%) and lower (25%) quartiles, open circles represent potential outliers. Levels of significance from Mann-Whitney U test denoted by **** ($p<0.0001$), *** ($p<0.005$), ** ($p=0.001$) and * ($p=0.01$).
